# Influence of Statins on Survival Outcome in Patients with Metastatic Castration Resistant Prostate Cancer Treated with Abiraterone Acetate

**DOI:** 10.1371/journal.pone.0161959

**Published:** 2016-09-01

**Authors:** Martin Boegemann, Katrin Schlack, Ann-Kathrin Fischer, Joachim Gerß, Julie Steinestel, Axel Semjonow, Andres Jan Schrader, Laura-Maria Krabbe

**Affiliations:** 1 Department of Urology, Muenster University Medical Center, Muenster, Germany; 2 Institute of Biostatistics and Clinical Research of the Westfalian Wilhelms-University Muenster, Schmeddingstraße 56, D-48149 Muenster, Germany; 3 Department of Urology, University of Texas Southwestern Medical Center, Dallas, Texas, United States of America; University of Minnesota Hormel Institute, UNITED STATES

## Abstract

**Objective:**

Even though the exact mechanism is largely unknown until now, statins are supposed to improve survival outcomes in various malignancies. For prostate cancer however, statins are known to compete with dehydroepiandrosterone (DHEAS) for the transport into the cytosol both using the cell by the Solute Carrier Transporter and thus diminish the cellular uptake of DHEAS as a precursor of androgens. Abiraterone inhibits CYP17A1 and thus effectively decreases the production of all relevant androgens including DHEAS. In this study we examined whether statins still affect survival outcome in patients with metastatic castration resistant prostate cancer (mCRPC) when treated with Abiraterone.

**Patients and Methods:**

108 men with mCRPC treated with Abiraterone from 02/2010 to 07/2015 with (n = 21) or without (n = 87) concomitant treatment with statins were investigated. Progression free survival (PFS) and overall survival (OS) were analyzed using Kaplan-Meier-estimates and univariate Cox-regression analysis. The influence on best clinical benefit under Abiraterone treatment was analyzed with bivariate and multivariate logistic regression analysis.

**Results:**

PSA-decline ≥ 50% was not significantly different in both groups (57 vs. 53%; p = 0.73). The median PFS (9 vs. 10 months; p = 0.97) and OS (14 vs. 18 months; p = 0.77) did not differ significantly between those men treated with and without concomitant statin therapy, respectively. Accordingly, there was no improvement for best clinical benefit in patients using statins (odds ratio: 1.2 (CI: 0.4–4.2); p = 0.76).

**Conclusion:**

Use of statins as concomitant medication did not improve survival outcomes or best clinical benefit in men with mCRPC treated with Abiraterone.

## Introduction

Statins, also known as 3-hydroxy-3-methylglutaryl-coenzyme A (HMG-CoA) reductase inhibitors, are originally used to treat hypercholesterolemia. However, next to inhibiting the synthesis of cholesterol, statins also lead to a decreased production of farnesyl pyrophosphate and geranyl pyrophosphate which both are essential for growth and proliferation of cells [1]. This and other potential effects of statins are part of the reason why statins are increasingly seen to harbour the capability to inhibit carcinogenesis and alter cancer outcomes in general apart from their protective effects on the cardiovascular system [2].

In prostate cancer another property of statins may add to their positive effect on survival. The solute carrier transporter (SLCO2B1) is an organic anionic transporter, which enables various anticancer compounds or hormones to enter cells [3]. Next to other substrates the adrenal androgen dehydroepiandrosterone (DHEAS), a precursor to the most potent androgen dihydroxytestosterone (DHT), which is the substrate binding and activating the androgen receptor in normal and PCa cells, is being transported into cells with the use of SLCO2B1. Following dedifferentiation and progression to castration resistant PCa (CRPC) the level of expression of SLCO2B1 increases [4]. Prior studies showed that the degree of response to androgen deprivation therapy (ADT) in patients with PCa is dependent on different variants of SLCO2B1 with different capability to perform androgen transportation into PCa cells [5, 6]. Other substrates of SLCO2B1 are statins. This may be part of the reason why the use of statins was generally associated with lower incidence and improved outcomes of PCa in earlier studies [7–10]. In a study on patients with hormone-sensitive PCa, Harshman et al. could recently show that the use of statins at the time of initiation of ADT was independently associated with significantly longer time to progression [11]. In addition to that, in the *in vitro*-part of the study, the group demonstrated that statins block the uptake of DHEAS competitively by binding to SLCO2B1, thus effectively decreasing the available intratumoral androgen pool and improving and prolonging the effect of primary ADT [11]. Intriguing findings like this have led to a subjectively felt increased use of statins as concomitant medication in men with hormone sensitive PCa even without presence of hypercholesterolemia.

Abiraterone is a potent 17α-hydroxylase / C17, 20-lyase (CYP17)-inhibitor that effectively blocks the synthesis of all relevant androgens, amongst others DHEAS, testosterone and DHT and has recently been approved for the treatment of metastatic castration resistant prostate cancer (mCRPC) in a setting prior to and after Docetaxel chemotherapy [12, 13]. This effect of Abiraterone on DHEAS questions the above named positive effect of statins on survival outcomes in mCRPC-patients treated with Abiraterone. Therefore, we aimed to investigate the impact on concomitant use of statins on best clinical benefit and survival outcomes in patients with mCRPC undergoing Abiraterone therapy.

## Patients and Methods

### Patients

We reviewed all patients with mCRPC out of a prospectively maintained who were treated with Abiraterone at the Department of Urology, Muenster University Medical Center, between 02/2010-07/2015. Best clinical benefit, progression free survival (PFS) and overall survival (OS) was evaluated with regard to concomitant statin use. The study was approved by the Institutional Review Board of the University of Muenster Medical Center and was conducted according to the principles expressed in the Declaration of Helsinki. Written informed consent has been obtained from the participants.

All patients presented with confirmed mCRPC as defined by prostate cancer working group 2 (PCWG2)-criteria for which metastatic PCa has to be progressing radiographically, clinically and/or biochemically with testosterone levels being in the castrate range [14]. All patients met the prerequisitions for Abiraterone-treatment in pre- or post-chemotherapy setting. The men receiving Abiraterone prior to Docetaxel chemotherapy had to be asymptomatic or oligo-symptomatic with no use of opiates and a pain level of ≤3 out of 10 on the numeric-rating-scale. Further, no visceral metastases were allowed to be evident. The patients who received Abiraterone in the post-chemotherapy setting (n = 47) all had progressive disease (PD) either on or after chemotherapy. Here, visceral metastases were allowed to be present. 61 patients were treated within the pre- chemotherapy setting. Five patients had received Enzalutamide prior to Abiraterone. All patients included in the analysis were either on a stable dose of a bone protective medication (Zoledronic acid or Denosumab) at least three months prior to start of Abiraterone and during the whole treatment phase or were not treated with bone protective medication at all.

Patients presented the day before start of Abiraterone-therapy to have blood drawn for baseline analysis of prostate specific antigen (PSA), two and four weeks after initiation of therapy and four-weekly thereafter. PSA-progression was defined according to PCWG2-criteria [14].

For baseline evaluation of soft tissue metastases CT-, or MRI-scans of thorax, abdomen and pelvis and for the acquisition of information on bone metastases bone scans were performed. Imaging was repeated during the course of the Abiraterone-therapy when clinically indicated but not in a routine fashion. PD was defined according to RECIST 1.1 criteria for cross sectional imaging [15] and by PCWG2 criteria for bone scans [14].

The current response status i.e. complete remission (CR), partial remission (PR), stable disease (SD) or PD was assessed at each visit. PD was declared upon deterioration of general condition or worsening of pain when unequivocally caused by prostate cancer, when PSA-progression according to PCWG2-criteria occurred, or when radiographic progression was seen.

The status of patients being concomitantly treated with statins or not was clarified by interviews directly before initiation of Abiraterone therapy. The patients were subdivided according to statin use (group-1: statin use; group-2: no statin use). All patients who received a statin used it beforehand to control dyslipidaemia and not for control of PCa.

### Statistical Methods

For descriptive statistics we report medians with 95% confidence intervals (CI) or interquartile range (IQR) for continuous variables and populations and frequencies for categorical variables. We determined the significance of the differences between categorical and continuous variables using the χ2-test, Fisher’s exact-test or Mann-Whitney U-test. For analysis of survival outcomes were applied Kaplan-Meier-estimates. We performed univariate and multivariate analysis with the use of binary logistic-regression for determining significance for best clinical benefit and of Cox-regression-models for survival outcomes. All reported p-values are two-sided and we assumed statistical significance when p was ≤0.05. We used SPSS-Statistics V.22 (IBM Inc., Armonk, NY, USA) for statistical assessment.

## Results

### Patient characteristics

Descriptive characteristics of the cohort are given in Table 1. The cohort consisted of 108 mCRPC patients; the median follow-up was 20.0 months (IQR, 11.0–28.0) for the patients alive at the time of analysis. The median time on treatment with Abiraterone was 10.0 months (IQR, 6.0–15.0). Fifteen (13.9%) men had ongoing therapy at the time of analysis. Dose modifications were only necessary for one patient for whom the Abiraterone dose had to be reduced by 50% due to elevation of transaminases after four weeks of therapy. The median age of the men in the entire cohort was 70.0 years (IQR, 62.3–76.8). Visceral metastases were found in 26.9% at the beginning of Abiraterone. An unfavorable initial Gleason-Score of ≥8 was seen in 60.2%. Median baseline PSA-, alkaline phosphatase (ALP)- and lactate dehydrogenase (LDH)-levels were 134 ng/ml (IQR, 44–371), 126 U/l (IQR, 86–297) and 252 U/l (IQR, 212–367), respectively.

**Table 1:** Baseline characteristics of patients with mCRPC on Abiraterone without or with statins and basic outcome data.

Variable		all	no statins	statins	p
**Baseline characteristics**					
**Patients [n], (%)**		108 (100)	87 (81)	21 (19)	-
**Median age [years] (IQR)**		70.0 (62.3–76.8)	70.0 (62.0–77.0)	71.0 (67.5–77.0)	0.487
**Lnn. Metastases [n] (%)**		68 (63.0)	55 (63.2)	13 (61.9)	0.911
**Visceral Metastases [n] (%)**		29 (26.9)	24 (23.8)	5 (23.8)	0.726
**Bone Metastases [n] (%)**		95 (88.0)	76 (87.4)	19 (90.5)	0.693
**Line of therapy [n] /%)**					
	**Pre CTX**	61 (56.5)	47 (54.0)	14 (66.7)	0.294
	**Post CTX**	47 (43.5)	40 (46.0)	7 (33.3)	
**Antiresorptive therapy [n] (%)**		62 (57.4)	48 (55.2)	14 (66.7)	0.339
	**Zoledronic acid**	41 (38.0)	30 (34.5)	11 (52.4)	0.129
	**Denosumab**	23 (21.3)	19 (21.8)	4 (19.0)	0.779
**ECOG (all) [n] (%)**					
	**0**	21 (19.6)	19 (22.1)	2 (9.5)	
	**1**	64 (59.8)	50 (58.1)	14 (66.7)	0.428
	**2**	22 (20.6)	17 (19.8)	5 (23.8)	
**GS ≥ 8 [n] (%)**		65 (60.2)	58 (66.7)	7 (33.3)	0.005
**Median PSA Baseline [ng/ml] (IQR)**		134 (44–371)	145 (46–457)	91 (30–206)	0.442
**Median LDH Baseline [U/l] (IQR)**		252 (212–367)	250 (206–369)	287 (234–420)	0.214
**Median ALP Baseline [U/l] (IQR)**		126 (86–297)	115 (84–285)	201 (115–478)	0.040
**LDH BL >UNL [n] (%)**		70 (64.8)	52 (59.8)	18 (85.7)	0.025
**Basic outcome data**					
**Median Follow-up [months] (IQR)**		20.0 (11.0–28.0)	18.0 (11.0–26.0)	29.0 (14.5–32.5)	0.120
**Median duration AA therapy [months] (IQR)**		10.0 (6.0–15.0)	10.0 (6.0–15.0)	9.0 (3.5-15-0)	0.329
**Patients died [n] (%)**		75 (69.4)	60 (69.0)	15 (71.4)	0.826
**Overall survival [months] (95%CI)**		16.0 (12.2–19.8)	18.0 (13.8–22.2)	14.0 (9.8–18.2)	0.770
**Best clinical outcome [n] (%)**					
	**CR**	1 (0.9)	0 (0)	1 (4.8)	
	**PR**	63 (58.9)	51 (59.3)	12 (57.1)	0.187
	**SD**	27 (25.2)	23 (26.7)	4 (19.0)	
	**PD**	16 (15.0)	12 (14.0)	4 (19.0)	
**PSA reduction ≥ 50% [n] (%)**		58 (53.7)	46 (52.9)	12 (57.1)	0.725
**PSA reduction ≥ 90% [n] (%)**		29 (26.9)	21 (24.1)	8 (38.1)	0.195
**ALP rising at 12 w AA [n] (%)**		25 (23.1)	21 (24.1)	4 (19)	0.620
**LDH normalization [n] (%)**		48 (44.4)	41 (47.1)	7 (33.3)	0.254

Abbreviations: IQR: Interquartile range; AA: Abiraterone; Lnn: Lymphonodal; CTX: Chemotherapy; CR: Complete remission; PR: Partial remission; SD Stable disease; PD: Progressive disease; ECOG: Eastern collaborative oncology group performance status; GS: Gleason score; PSA: Prostate specific antigen; LDH: Lactate dehydrogenase; ALP: Alkaline Phophatase; BL: Baseline; UNL: Upper normal limit; 95%CI: 95% Confidence interval

Concomitant statin medication was present in 21 (19%) patients. Patients with statin medication had significantly higher baseline median ALP than patients without statin use (201 vs. 115 U/l; p = 0.04 (Mann-Whitney U-test)), a larger proportion of patients with LDH-levels above the upper limit of normal (85.7 vs. 59.8%; p = 0.03, (χ^2^-test)) and a smaller proportion of a Gleason score ≥8 (33.3 vs. 66.7%, p = 0.01, (χ^2^-test)). All other baseline characteristics, including the use of bone protective medication (66.7 vs. 55.2%), did not differ significantly.

**Fig 1:**
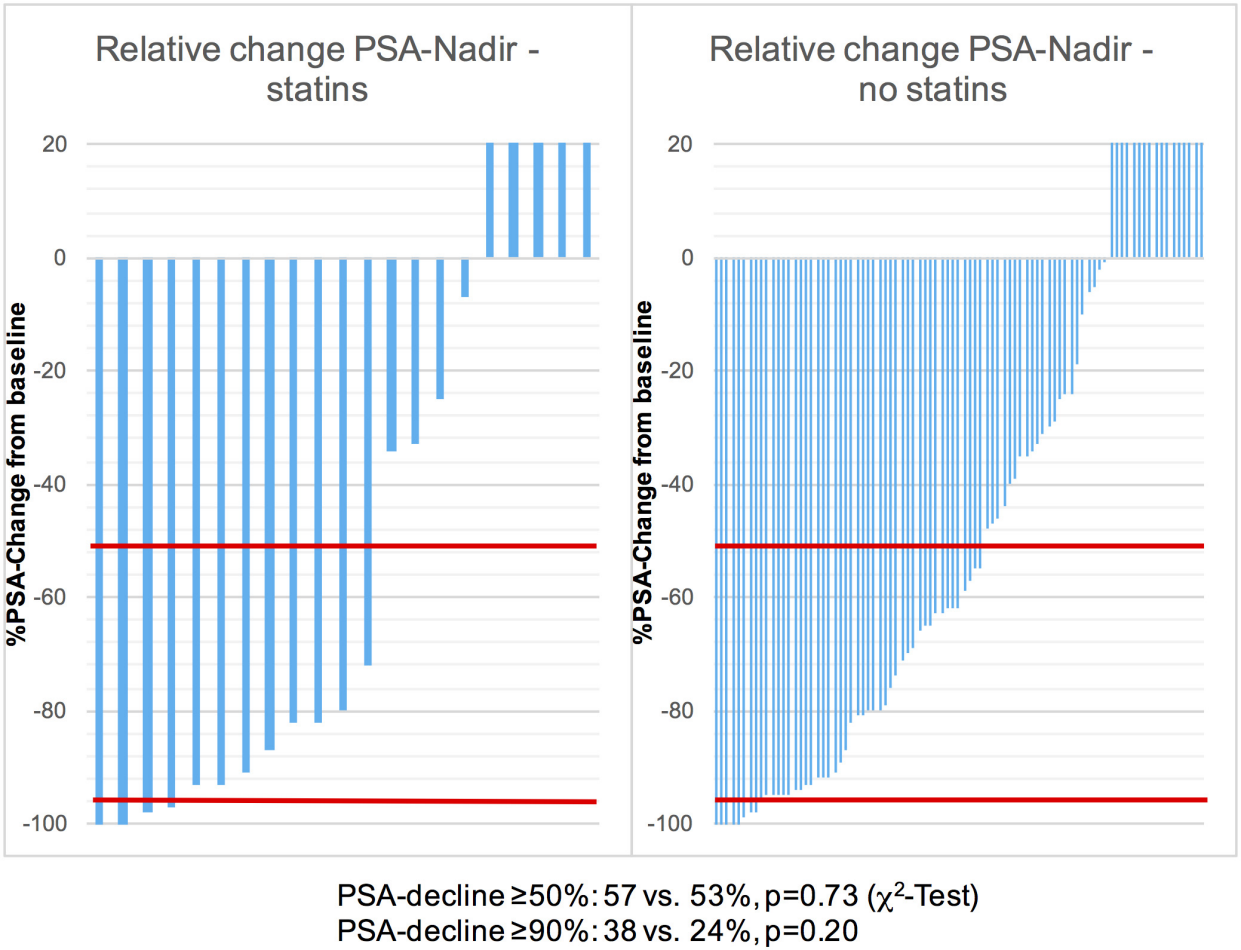
Waterfall-Plots of relative PSA-Nadir with or without concomitant statin-use in mCRPC patients on Abiraterone therapy. In the subpopulations of patients with or without concomitant statin use the PSA-decline of ≥50% occurred in 57 and 53% of patients. A decline of ≥90% was seen in 38 and 24%. The differences were non-significant (p = 0.73 and 0.20, respectively). The red lines represent a PSA-decline of 50 and 90%, respectively. Patients with rising PSA-values only were censored at 20% PSA-incline.

PSA decline of ≥50% was seen in 57.1% of the patients treated with statins and in 52.9% treated without statins (p = 0.73, χ^2^-test). A decline of ≥90% was found in 38.1 vs. 24.1% of the patients (p = 0.20, χ^2^-test) (Fig 1).

In the follow-up period after progression on Abiraterone the following subsequent treatments were given to the patients: Enzalutamide (35%), Docetaxel (25% (only patients with Abiraterone prior to chemotherapy)), Cabazitaxel (4%), Radium-223 (7%) and ^177^Lutetium-PSMA-Ligand therapy (4%).

### Impact of statin use on best clinical benefit and survival

In univariate analyses for best clinical benefit (defined as CR/PR/SD vs. PD) concomitant treatment with statins was not associated with an improved benefit in patients on Abiraterone. The odds ratio was 1.2 for patients with statin use regarding this endpoint (95% confidence interval (95%CI): 0.4–4.1; p = 0.76). The status of metastases and the line of Abiraterone therapy did all not significantly influence best clinical benefit (Table 2).

**Table 2:** Univariate Cox-regression analysis for overall survival and progression free survival and bivariate regression analysis for the prediction of best clinical benefit in 108 patients treated with Abiraterone with or without statins as concomitant medication.

**Overall Survival**			
**Variable**		**HR (95% CI)**	**p**
**Use of statins**			0.77
	**No**	1 (reference)	
	**Yes**	1.1. (0.6–1.9)	
**Abiraterone**			0.04
	**Pre Docetaxel**	1 (reference)	
	**Post Docetaxel**	1.6 (1.0–2.6)	
**Lymphonodal metastases**			0.23
	**No**	1 (reference)	
	**Yes**	0.8 (0.5–1.2)	
**Visceral Metastases**			0.63
	**No**	1 (reference)	
	**Yes**	1.1 (0.7–1.9)	
**Bone Metastases**			0.76
	**No**	1 (reference)	
	**Yes**	1.1 (0.5–2.6)	
**Odds for best clinical benefit**			
**Variable**		OR (95% CI)	P
**Use of statins**			0.76
	**No**	1 (reference)	
	**Yes**	1.2 (0.4–4.1)	
**Abiraterone**			0.57
	**Pre Docetaxel**	1 (reference)	
	**Post Docetaxel**	1.3 (0.5–3.7)	
**Lymphonodal metastases**			0.70
	**No**	1 (reference)	
	**Yes**	1.2 (0.4–3.6)	
**Visceral Metastases**			0.22
	**No**	1 (reference)	
	**Yes**	1.9 (0.7–5.6)	
**Bone Metastases**			0.52
	**No**	1 (reference)	
	**Yes**	0.6 (0.2–2.6)	
**Progression Free Survival**			
**Variable**		**HR (95% CI)**	**p**
**Use of statins**			0.97
	**No**	1 (reference)	
	**Yes**	1.0 (0.6–1.7)	
**Abiraterone**			0.05
	**Pre Docetaxel**	1 (reference)	
	**Post Docetaxel**	1.5 (1.0–2.3)	
**Lymphonodal metastases**			0.65
	**No**	1 (reference)	
	**Yes**	0.9 (0.6–1.4)	
**Visceral Metastases**			<0.01
	**No**	1 (reference)	
	**Yes**	1.9 (1.2–2.9)	
**Bone Metastases**			1.00
	**No**	1 (reference)	
	**Yes**	1.0 (0.5–1.9)	

Abbreviations: HR: Hazard ratio; 95% CI: 95% Confidence interval; OR: Odds ratio

Results of univariate analyses for PFS and OS are displayed in Table 2. Again, concomitant statin use was not associated with improved survival outcomes. For PFS- and OS-analysis the hazard ratios (HR) for no use of statins was 1.0 (95%CI: 0.6–1.7; p = 0.97) and 1.1 (95%CI: 0.6–1.9; p = 0.77), respectively. Treatment with Abiraterone post chemotherapy was associated with shorter PFS (HR: 1.5, 95%CI: 1.0–2.3; p = 0.05) as well as OS (HR: 1.6, 95%CI: 1.0–2.6; p = 0.04) and presence of visceral metastases was a significant prognosticator for worse PFS (HR: 1.9, 95%CI: 1.2–2.9; p<0.01). All other clinical parameters had no significant impact on survival outcomes.

**Fig 2:**
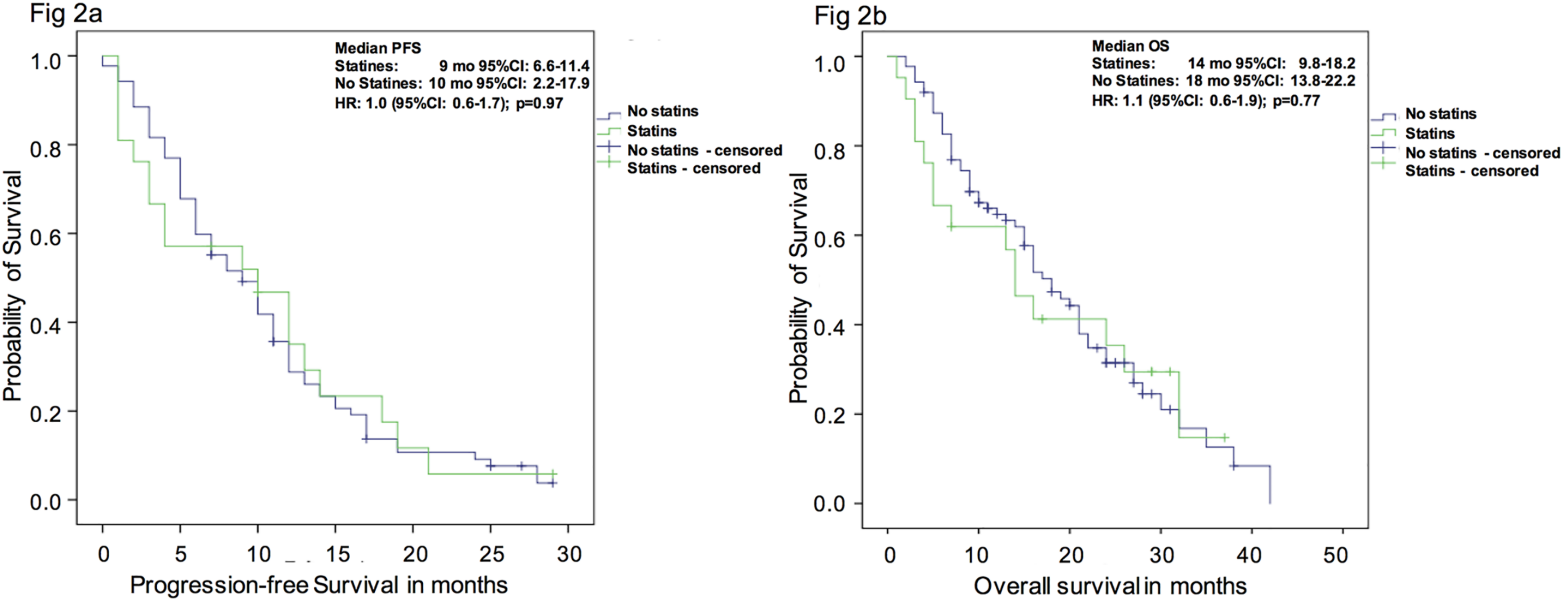
a) Progression free and b) overall survival probability of patients with mCRPC under therapy with Abiraterone with or without concomitant statin use. The Kaplan-Meier analysis of PFS (Fig 2a) and OS (Fig 2b) showed non-significant differences between mCRPC patients treated with Abiraterone with or without concomitant use of statins.

Concordant with the regression analyses, the Kaplan-Meier estimates showed no significant differences in median survival both for PFS and OS (Fig 2). For PFS the median survival of patients with statin use was 9 months (95%CI: 6.6–11.4 months), as compared to 10 months in patients without use of statins (95%CI: 2.2–17.9 months; log-rank, p = 0.97). For OS-analysis the median survival for men with concomitant statin use was 14 months (95%CI: 9.8–18.2 months) compared to 18 months (95%CI: 13.8–22.2 months) in patients without statins (log-rank, p = 0.77).

After multivariate adjustment with clinically important covariates (status of metastases and line of therapy), the concomitant use of statins remained not associated with improved survival outcomes or best clinical benefit. Only use of Abiraterone post chemotherapy and presence of visceral metastases were independent prognosticators of worse progression free survival (Table 3).

**Table 3:** Multivariate Cox-regression analysis for overall survival and progression free survival and bivariate regression analysis for the prediction of best clinical benefit (PD vs. CR/PR/SD) in 108 patients treated with Abiraterone with or without statins as concomitant medication.

**Overall Survival**			
**Variable**		**HR (95% CI)**	**p**
**Use of statins**			0.63
	**No**	1 (reference)	
	**Yes**	1.2. (0.7–2.1)	
**Abiraterone**			0.07
	**Pre Docetaxel**	1 (reference)	
	**Post Docetaxel**	1.6 (1.0–2.7)	
**Lymphonodal metastases**			0.54
	**No**	1 (reference)	
	**Yes**	0.9 (0.5–1.4)	
**Visceral Metastases**			0.60
	**No**	1 (reference)	
	**Yes**	1.2 (0.7–1.9)	
**Bone Metastases**			0.76
	**No**	1 (reference)	
	**Yes**	0.9 (0.4–2.1)	
**Odds for best clinical benefit**			
**Variable**		OR (95% CI)	p
**Use of statins**			0.63
	**No**	1 (reference)	
	**Yes**	1.4 (0.4–4.9)	
**Abiraterone**			0.44
	**Pre Docetaxel**	1 (reference)	
	**Post Docetaxel**	1.5 (0.5–4.6)	
**Lymphonodal metastases**			0.79
	**No**	1 (reference)	
	**Yes**	1.2 (0.4–3.7)	
**Visceral Metastases**			0.25
	**No**	1 (reference)	
	**Yes**	1.9 (0.6–5.5)	
**Bone Metastases**			0.46
	**No**	1 (reference)	
	**Yes**	0.6 (0.1–2.6)	
**Progression Free Survival**			
**Variable**		**HR (95% CI)**	**p**
**Use of statins**			0.83
	**No**	1 (reference)	
	**Yes**	1.1 (0.6–1.8)	
**Abiraterone**			0.05
	**Pre Docetaxel**	1 (reference)	
	**Post Docetaxel**	1.6 (1.0–2.5)	
**Lymphonodal metastases**			0.75
	**No**	1 (reference)	
	**Yes**	0.9 (0.6–1.5)	
**Visceral Metastases**			<0.01
	**No**	1 (reference)	
	**Yes**	1.9 (1.2–3.1)	
**Bone Metastases**			0.55
	**No**	1 (reference)	
	**Yes**	0.8 (0.4–1.6)	

Abbreviations: HR: Hazard ratio; 95% CI: 95% Confidence interval; OR: Odds ratio

## Discussion

Almost all PCa initially respond to ADT. However, in the end continuous ADT inevitably leads to development of CRPC. One reason for this development is that androgen receptor signaling can still be triggered by residual androgens even if serum testosterone levels are reduced below castration limits [16–18]. DHEAS is a precursor of more potent androgens and competes with statins to be transported into the cytosol of the cell using the organic anionic transporter SLCO2B1 [3]. Prior work has shown that response to ADT depends on variants of SLCOB1 which have different capability of transporting DHEAS into cells [5, 6]. Harshman et al. demonstrated *in vitro* that statins diminish DHEAS-stimulated proliferation of hormone sensitive PCa cells [11]. In the same trial they showed that patients treated with statins at the time of initiation of ADT and beyond, had prolonged time to progression compared to patients without concomitant use of statins.

These results are in line with epidemiological studies that mostly showed significant associations between the use of statins and lower PSA-levels, reduced incidence of clinically significant and advanced PCa, decreased recurrence rates after local treatment as well as better survival outcome [7, 9, 19–21]. A large meta-analysis revealed that the use of statins can decrease the development of any PCa by 7%, and all but one of the studies in this meta-analysis showed a relative risk reduction of developing clinically significant or advanced PCa [7].

However, an *in vitro* study on different PCa cell lines including CRPC (castration resistant LNCaP subtype and VCaP) by Murtola et al. demonstrated that statins inhibited only hormone-sensitive but not the CRPC cell lines [22]. Reasons for this may be that that in CRPC very little residual androgen activity is sufficient to keep the androgen receptor axis going or the occurrence of intratumoral androgen production [16–18]. Another underlying cause for this phenomenon is the re-establishing of androgen receptor signalling by promiscuity of the receptor which can be activated by ligands other than androgens, for example progesterone, corticosteroids or sometimes even medication like antiandrogens [23, 24]. Specifically in patients treated with Abiraterone, maintenance of androgens in the tumour cells can be sustained by overexpression of CYP17A1 [25]. All of these effects make an effect of statins on the available intratumoral androgen pool unlikely.

In clinical routine statins are effectively used to treat hypercholesterolemia by inhibiting the HMG-CoA-reductase. Androgens are steroid hormones and the basic component of all androgens is cholesterol. So by reducing the available cholesterol in PCa cells with accelerated androgen metabolism and therefore increased need for cholesterol, statins may preclude proliferative activity of PCa. The impact of statins on the SLCO2B1-transporter may add to this potential effect.

Abiraterone is a very potent CYP-17-Inhibitor and subtotally reduces the levels of all relevant androgens, including DHEAS. Therefore, the effect of statins on the SLCO2B1-transporter and thus reducing the levels of DHEAS might not be of additional value in patients receiving Abiraterone [26].

Statins have the reputation of being rather non-toxic. However, there are some important caveats. In susceptible patients with risk factors for developing diabetes mellitus, statins can hasten the progression to manifest diabetic disease [27]. The use of prednisone as an obligate co-medication next to Abiraterone, which by itself poses an increased risk of *de novo* diabetes mellitus, may even increase this risk. Furthermore, statins increase the risk of muscle-related side effects. These side effects occur in 5–10% of patients treated with statins [28]. However, the most serious effect of statins, rhabdomyolysis, is an exceedingly rare event. In addition to these adverse events drug interactions need to be kept in mind. Like Abiraterone, statins are substrates for CYP3A4, therefore an unfavourable interaction per se is unlikely. However, when combined with CYP3A4 inducers the effects of Abiraterone and/or statins may be unforeseeable.

As the recent reports of the potentially PCa-protective effects of statins get to the public domain and due to the alleged favourable toxicity profile of statins, more and more men with PCa are seemingly treated with statins to improve their respective outcome of PCa even when no hypercholesterolemia is evident.

In our study we found that in patients treated with Abiraterone the concomitant use of statin medication did not improve the best clinical benefit, progression-free survival or overall survival. Owing to the method of action of Abiraterone with subtotal depression of cholesterol dependent steroid-androgens, including DHEAS, our results support the hypothesis that the effect of statins to reduce the available cholesterol pool and of interfering with the SLCO2B1-transporter adds no further to the effect caused by Abiraterone alone.

Our study is limited by the problems inherent to its retrospective approach even in the setting of a prospectively maintained database. We did not collect statin specific toxicity data. Statin users may represent a population that cares more about their individual health and therefore might bias our results. Finally, we did not stratify between different available statins with potentially different potency and pharmacokinetic impact that could have influenced the extent of our findings.

Despite these limitations, our study is the first that relates to the impact of statins on survival outcomes in patients treated with next generation ADT in CRPC and suggests caution with respect to the application of statins in patients without coincidental hypercholesterolemia.

## Conclusions

In mCRPC patients treated with Abiraterone statins as concomitant medication do not seem to improve the best clinical benefit under Abiraterone and do not improve survival outcomes. Overdosing of statins or interaction with concomitant medication can lead to severe side effects. Therefore, the administration of statins should be critically reviewed in CRPC patients treated with Abiraterone in case of normocholesterolemia.

## Supporting Information

S1 File Basic Data Set: SPSS raw data. (SAV)

## Abbreviations

DHEAS: Dehydroepiandrosteron
SLC-Transporter: Solute Carrier Transporter
mCRPC: Metastatic castration resistant prostate cancer
PFS: Progression free survival
OS: Overall survival
HR: Hazard ratio
HMG-CoA: 3-hydroxy-3-methylglutaryl-coenzyme A
95%CI: 95% Confidence interval
DHT: Dihydroxytestosterone
ADT: Androgen deprivation therapy
CYP17: 17α-hydroxylase / C17, 20-lyase
PCWG2: Prostate cancer working group 2
PD: Progressive disease
PSA: Prostate specific antigen
CR: Complete remission
PR: Partial remission
SD: Stable disease
IQR: Interquartile range
ALP: Alkaline phosphatase
LDH: Lactate dehydrogenase
